# Supplementary material for: A Dynamic Tool to Describe Lamb Growth and Its Use as a Decision Support System
Source: Animals (Basel). 2024 Aug 2;14(15):2246. doi: 10.3390/ani14152246 (PMC11311011; doi:10.3390/ani14152246)
Supplement: Supplementary file 1 [file animals-14-02246-s001.zip › animals-3060122-supplementary.pdf]

Supplementary Materials

A Dynamic Tool to Describe Lamb Growth and Its Use as a Decision Support System

Table S1. Evaluation dataset.

Reference	Treatment	N	Breed	SFW[a] (kg)	Sex	ADF (%)	NDF (%)	CP (%)	ME (Mcal/kg)	ADG (kg)	Observed DMI (kg/day)	Initial age (days)	Days on feed	IBM[b] (kg)	OFBM[c] (kg)	PFBM[d] (kg)
Lopez-Carlos et al. (2011)	Control	10	Dorper x Katadhin	105	Male	31.70	52.40	18.20	2.92	0.233	1.440	75	42	28.40	38.20	39.82
Papi et al. (2011)	Concentrate 30%	20	Fat tailed chall	80	Male	20.80	36.80	14.30	2.17	0.244	2.330	165	84	38.90	59.40	55.25
	Concentrate 50%	20	Fat tailed chall	80	Male	16.80	33.60	15.20	2.38	0.269	2.280	165	84	39.10	61.70	57.66
	Concentrate 70%	20	Fat tailed chall	80	Male	11.20	24.80	16.10	2.55	0.278	2.080	165	84	37.70	61.00	58.27
	Concentrate 90%	20	Fat tailed chall	80	Male	7.20	22.60	17.40	2.71	0.238	1.750	165	84	38.00	58.00	60.09
Sheridan et al. (2003)	High energy	8	Mutton Merino	105	Male	17.78	35.92	14.56	2.89	0.281	1.780	90	56	33.31	49.05	51.43
	Low energy	8	Mutton Merino	105	Male	24.74	44.17	14.29	2.36	0.203	1.890	90	56	32.13	43.51	44.45
Cunha et al. (2008)	Cotton seed 0%	6	Santa Ines	100	Male	19.80	36.40	15.00	2.39	0.206	1.226	120	70	19.10	33.50	28.18
	Cotton seed 20%	6	Santa Ines	100	Male	24.90	41.60	15.00	2.41	0.186	1.236	120	70	19.16	32.66	28.45
	Cotton seed 30%	6	Santa Ines	100	Male	30.10	49.60	15.90	2.36	0.149	1.126	120	70	19.96	30.41	29.44
	Cotton seed 40%	6	Santa Ines	100	Male	31.70	49.60	16.70	2.40	0.174	1.920	120	70	19.96	32.16	29.77
Azevedo et al. (2012)	Macauba 0%	6	Santa Ines	100	Male	16.75	32.09	15.01	2.39	0.205	1.094	150	60	23.55	35.86	33.67
	Macauba 10%	6	Santa Ines	100	Male	20.33	36.35	15.88	2.36	0.200	1.093	150	60	24.18	36.16	34.14
	Macauba 20%	6	Santa Ines	100	Male	24.96	40.88	15.81	2.33	0.203	1.133	150	60	23.65	35.85	33.33
	Macauba 30%	6	Santa Ines	100	Male	27.23	43.17	15.74	2.31	0.188	1.179	150	60	24.11	35.38	33.87
Ebrahimi et al. (2007)	High energy	18	Mehraban	81	Male				2.70	0.219	1.500	210	70	35.40	50.70	54.73
	Medium energy	18	Mehraban	81	Male				2.50	0.203	1.400	210	70	35.40	49.60	52.81
	Low energy	18	Mehraban	81	Male				2.30	0.119	1.200	210	70	35.30	43.60	50.73

Kioumarsi et al. (2008)	Low energy 12% CP	6	Taleshi	35	Male			12.00	2.30	0.111	1.108	165	105	19.70	30.02	29.84
	Low energy 14% CP	6	Taleshi	35	Male			14.00	2.30	0.098	1.109	165	105	19.70	29.15	29.84
	Low energy 16% CP	6	Taleshi	35	Male			16.00	2.30	0.112	1.141	165	105	19.70	29.38	29.84
	High energy 12% CP	6	Taleshi	35	Male			12.00	2.50	0.129	1.171	165	105	19.70	30.02	30.52
	High energy 14% CP	6	Taleshi	35	Male			14.00	2.50	0.148	1.200	165	105	19.70	33.44	30.52
	High energy 16% CP	6	Taleshi	35	Male			16.00	2.50	0.146	1.211	165	105	19.70	33.28	30.52
Alves et al. (2003)	Low energy	6	Santa Ines	100	Male	29.40	58.30	18.48	2.42	0.123	0.900	180	98	20.17	31.87	36.17
	Medium energy	6	Santa Ines	100	Male	21.39	44.23	18.51	2.66	0.137	0.870	180	96	20.10	31.10	39.00
	High energy	6	Santa Ines	100	Male	13.26	29.96	18.48	2.83	0.191	0.870	180	76	20.47	31.90	36.30
Pereira et al. (2008)	Citrus 0%	6	Santa Ines	100	Male	22.77	43.36	15.11	2.54	0.245	1.098	90	67	18.74	31.70	28.14
	Citrus 25%	6	Santa Ines	100	Male	22.75	39.62	15.16	2.58	0.271	1.159	90	67	18.05	32.42	27.18
	Citrus 50%	6	Santa Ines	100	Male	22.74	35.88	15.20	2.62	0.305	1.289	90	67	18.82	34.97	28.89
	Citrus 75%	6	Santa Ines	100	Male	22.73	32.15	15.27	2.77	0.274	1.173	90	67	18.15	32.65	28.75
Haddad and Younis (2004)	Control	7	Awassi	80	Male	14.00	27.00	15.50	2.72	0.202	0.986	65	72	20.30	34.90	35.62
	Low fat	7	Awassi	80	Male	14.00	26.00	15.40	2.84	0.205	0.901	65	72	20.50	35.10	37.13
Fimbres et al. (2002)	Forage 0%	5	Pelibuey	55	Male	CF[h]	CF	15.00	2.96	0.250	0.960	90	60	23.90	39.00	37.75
	Forage 10%	5	Pelibuey	55	Male	CF	CF	15.00	2.88	0.207	1.200	90	60	23.90	36.30	37.32
	Forage 20%	5	Pelibuey	55	Male	CF	CF	15.00	2.78	0.203	1.377	90	60	23.90	36.50	36.77
	Forage 30%	5	Pelibuey	55	Male	CF	CF	15.00	2.61	0.174	1.429	90	60	23.90	31.10	35.83
Bensimon et al (2011)	Glycerin 0%	7	Santa Ines	100	Male	22.70	9.65	17.04	2.64	0.210	1.260	90	44	26.07	34.57	35.53
	Glycerin 15%	10	Santa Ines	100	Male	21.40	9.72	17.16	2.61	0.240	1.300	90	43	26.19	35.65	35.08
	Glycerin 30%	10	Santa Ines	100	Male	18.56	10.13	16.92	2.58	0.230	1.270	90	47	26.49	35.82	36.49
Sadri et al. (2018)	PWSS[e] 0%	6	Mehraban	81	Male	15.50	28.20	16.60	2.63	0.220	1.158	135	60	25.00	38.20	38.99
	PWSS 10%	6	Mehraban	81	Male	15.50	27.40	16.60	2.63	0.221	1.106	135	60	24.90	38.20	38.85
	PWSS 20%	6	Mehraban	81	Male	15.50	26.70	16.60	2.63	0.222	1.067	135	60	24.90	38.30	38.85
	PWSS 3 %	6	Mehraban	81	Male	15.60	26.00	16.60	2.63	0.239	1.108	135	60	25.00	40.10	38.99
Jaborek et al. (2016)	Metal bed	24	Traghee Hampshire	119	Male			15.31	2.62	0.258	1.232	100	85	35.21	57.29	60.19

	Sand bed	24	Traghee Hampshire	119	Male			15.31	2.62	0.273	1.250	100	84	35.18	58.17	59.80
	Straw bed	24	Traghee Hampshire	119	Male			15.31	2.62	0.285	1.281	100	83	35.29	58.96	59.63
Estrada-Angulo et al. (2018)	Crude Protein 11%	10	PelibueyxKatahdin	75	Male		15.40	11.10	2.80	0.242	1.049	90	84	23.95	43.35	45.62
	Crude Protein 14%	10	PelibueyxKatahdin	75	Male		16.50	14.10	2.80	0.266	1.136	90	84	24.04	45.42	45.75
	Crude Protein 17%	10	PelibueyxKatahdin	75	Male		16.90	16.90	2.80	0.276	1.150	90	84	24.05	46.27	45.76
	Crude Protein 20%	10	PelibueyxKatahdin	75	Male		17.10	19.80	2.80	0.300	1.230	90	84	23.94	48.22	45.61
Costa et al. (2012)	Cactus pear 0%	9	Santa Ines	100	Male		42.60	16.20	2.30	0.255	1.300	120	35	26.60	35.90	32.44
	Cactus pear 25%	9	Santa Ines	100	Male		43.90	15.90	2.20	0.236	1.400	120	35	27.30	35.80	32.85
	Cactus pear 50%	9	Santa Ines	100	Male		45.30	15.50	2.20	0.218	1.500	120	35	28.00	35.90	33.71
	Cactus pear 75%	9	Santa Ines	100	Male		46.70	15.20	2.10	0.231	1.400	120	35	27.70	36.00	32.86
	Cactus pear 100%	9	Santa Ines	100	Male		48.10	14.90	2.10	0.210	1.300	120	35	27.90	35.20	33.10
Tadayon et al. (2017)	RPB[f] 0% DOP[g] 0%	6	Fat tailed chall	80	Male	19.2	28.80	14.62	2.49	0.208	1.266	120	60	28.00	41.60	41.81
	RPB 0% DOP 110%	6	Fat tailed chall	80	Male	20.10	29.00	14.69	2.48	0.227	1.407	120	60	27.90	42.70	41.60
	RPB 0% DOP 220%	6	Fat tailed chall	80	Male	21.60	29.10	14.72	2.50	0.261	1.447	120	60	28.20	45.20	42.15
	RPB 160% DOP 0%	6	Fat tailed chall	80	Male	19.30	27.30	14.52	2.47	0.237	1.387	120	60	28.00	43.40	41.64
	RPB 160% DOP 110%	6	Fat tailed chall	80	Male	20.40	27.40	14.62	2.50	0.278	1.403	120	60	28.20	46.20	42.15
	RPB 1600% DOP 200%	6	Fat tailed chall	80	Male	21.90	28.80	14.59	2.49	0.297	1.507	120	60	28.00	48.50	41.81

[a] Standard final weight, [b] Initial body mass, [c] Observed final body mass, [d] Predicted final body mass, [e] Potato-wheat straw silage, [f] Recycled poultry bedding, [g] Dried orange pulp, and [h] Crude fiber. The Dynamic Lamb Growth Model can be downloaded at: https://bit.ly/3V1uVxA ; How to use the Dynamic Lamb Growth Model: bit.ly/4aBE31C.
